# Clinical characteristics and risk factors of nonalcoholic fatty liver disease in children with obesity

**DOI:** 10.1186/s12887-021-02595-2

**Published:** 2021-03-12

**Authors:** Luting Peng, Su Wu, Nan Zhou, Shanliang Zhu, Qianqi Liu, Xiaonan Li

**Affiliations:** 1 Department of Children Health Care, Children’s Hospital of Nanjing Medical University, 72 Guangzhou Road, Nanjing, 210008 People’s Republic of China; 2 Department of Endocrinology, Children’s Hospital of Nanjing Medical University, Nanjing, 210008 People’s Republic of China; 3 Department of Ultrasonography, Children’s Hospital of Nanjing Medical University, Nanjing, 210008 China; 4 Institute of Pediatric Research, Nanjing Medical University, Nanjing, 210008 China

**Keywords:** Obese children, Nonalcoholic fatty liver disease, Nonalcoholic steatohepatitis, Gender, Uric acid, Insulin resistance

## Abstract

**Background:**

With the increasing number of children with obesity worldwide, nonalcoholic fatty liver disease (NAFLD) has become the most common liver disease among children. It is necessary to recognize the risk factors of NAFLD for prevention in childhood since NAFLD is asymptomatic in the early stage. *Objectives.* The objective of this study was to investigate possible risk factors of NAFLD in children with obesity, providing evidence for monitoring and prevention strategies at an early stage for obese children with NAFLD.

**Methods:**

Data were collected from 428 children and adolescents aged 6-16 years recruited from the Children’s Hospital at Nanjing Medical University from September 2015 to April 2018 and analyzed. Based on a combination of ultrasound results and alanine transaminase levels, subjects were divided into three groups: simple obesity (SOB), simple steatosis (SS), and nonalcoholic fatty hepatitis (NASH). Blood biochemical examination included glucose, insulin, uric acid, lipid profile and liver enzymes.

**Results:**

Among 428 children with obesity, 235 (54.9%) had SS and 45 (10.5%) had NASH. Body mass index, body mass index standard deviation score (BMI-SDS), waist circumference, body fat, liver enzymes, uric acid and HOMA-IR level were significantly higher in the NASH group than in the SS and SOB groups (*p* < 0.001). 53.3% of the SS group and 49.8% of the NASH group had metabolic syndrome, significantly more than in the SOB group (19.6%, *p* < 0.001). After adjustment for confounding factors, logistic regression models revealed that NASH was associated with BMI-SDS ≥ 3, gender, hyperuricemia and insulin resistance.

**Conclusions:**

The prevalence of NASH in children with obesity is closely related to high BMI-SDS, gender, insulin resistance and hyperuricemia. These findings provide evidence that monitoring risk factors of childhood obesity can assist in developing prevention strategies for liver disease at an early stage.

## Background

With childhood obesity rates increasing, nonalcoholic fatty liver disease (NAFLD) has become the most common liver disease among children worldwide [1]. The prevalence of NAFLD in Chinese children was 3.4% [2]. The prevalence in obese and overweight children was significantly higher, ranging from 50 to 80% [3]. NAFLD may progress from simple steatosis (SS) to nonalcoholic steatohepatitis (NASH), subsequently leading to fibrosis/cirrhosis [4, 5]. Among those obese youth with NAFLD, 10% have NASH, characterized by hepatocyte inflammation and expansion in the background of hepatic steatosis [1, 6–8]. Although simple hepatic steatosis usually has a “benign procedure”, NASH may degenerate into end-stage liver disease. Children can develop to a harmful stage faster than adults [9]. Liver cirrhosis due to NAFLD in children has been described [10].

In addition to intrahepatic lesions, NAFLD also has serious health consequences beyond the liver associated with metabolic disorder, cardiovascular disease and insulin resistance [11, 12]. Among patients with nonalcoholic steatohepatitis, half of the deaths were due to cardiovascular disease and malignancy [13, 14]. Therefore, early identification of SS and NASH is crucial for treatment and prognosis.

Liver biopsy is considered the gold standard for the diagnosis of NAFLD, which can facilitate the differentiation of SS and NASH in both children and adults [15]. However, it is not suitable for screening among children because of its invasive nature and cost. In addition, only a small portion of the liver is examined, which may lead to sample errors and selection bias. A noninvasive and qualitative alternative is to examine steatosis of the liver by ultrasound [15]. In addition, blood alanine transaminase (ALT) is an inexpensive, minimally invasive, acceptable and universally available blood test. NAFLD is the most common cause of elevated liver enzymes in patients in developed countries. NASH is associated with a two-fold increase in ALT in children with obesity [15, 16].

Studies in adults have shown the link between NAFLD and metabolic disorders [17]. Longitudinal studies in adults demonstrate that patients with NAFLD have increased incidence of diabetes, metabolic syndrome and mortality compared with matched control populations [18]. However, studies on the associations between epidemiological situations, risk factors and cardiovascular risk of NAFLD in obese children are few, especially in China. Children are in a rapid growth and development period, and their pathophysiological changes are different from those of adults. Today’s children with obesity are also exposed to maternal obesity and insulin resistance earlier than those decades ago. Like other liver diseases, NAFLD is asymptomatic in the early stage, and can easily go unnoticed by clinicians. This clinical study aimed to investigate possible risk factors of NAFLD in obese children in the Chinese population, specifically the city of Nanjing.

## Methods

### Subjects and inclusion criteria

This study was performed in Nanjing, China, at the Children’s Hospital of Nanjing Medical University. The study protocol was approved by the Medical Ethics Committee of the Children’s Hospital of Nanjing Medical University (201412004.1). Children from 6 to 16 years old were recruited to the study from September 2015 to April 2018. The study was conducted in obese children. Inclusion criterion was body mass index (BMI) ≥95th percentile according to China’s children’s BMI classification. Exclusion criteria were any other endocrine diseases, viral hepatitis, hereditary diseases and other infectious or chronic diseases.

Pubertal stage was evaluated on the basis of the Tanner scale. Children with obesity were further divided into three subgroups by combining ultrasound and ALT 2-fold elevation: simple obese (SOB) group with normal liver in ultrasound, simple steatosis (SS) group with fatty liver in ultrasound and ALT < 80 U/L, and NASH group with fatty liver in ultrasound and ALT ≥80 U/L, according to the two-fold lab reference standard for impaired liver function [15].

Study staff gave oral and written information to parents/caregivers. The parent(s) or caregiver(s) of all subjects provided written informed consent before inclusion in the study. Written assent was obtained for all children. Through interview with children and their parents, experienced interviewers collected the basic information about the children, including birth history, family history and lifestyles.

### Anthropometric measurements

All participants had anthropometric assessments. Experienced researchers measured height and weight using standardized measuring methods [19]. A digital scale (graduation 100 g) was used for weight measurement, and a gauge (graduation 1 mm) (Seca 704, Germany) for height measurement. BMI was calculated by dividing body weight (in kilograms) by the square of height (in meters). BMI standard deviation score (BMI-SDS) was calculated according to WHO reference values. At the end of the expiratory period, the waist circumference was measured at the navel with a non-retractable soft ruler to the nearest 0.1 cm [20]. Waist circumference (in centimeters) was divided by height (in centimeters) to calculate the waist to height ratio (WHtR). Body fat and skeletal muscle were measured by InBody J20 (Biospace, Korea). Percentage of body fat and percentage of skeletal muscle were calculated. After a quiet rest for 10 min, blood pressure was measured three times by an electronic sphygmomanometer (Omron HBP-1300) and the mean value was taken.

### Blood biochemical examination

All subjects were told to fast overnight for 12 h before phlebotomy the next morning. Fasting laboratory assays included glucose, insulin, total cholesterol, high-density lipoprotein (HDL) cholesterol, triglycerides, alanine aminotransferase (ALT), and aspartate aminotransferase (AST).

### Abdominal ultrasound examination

All subjects were examined after 12 h of fasting overnight. Hepatic ultrasonography was performed by ultrasound technician to establish a diagnosis according to at least two of the following criteria: higher echogenicity higher in the liver than in the spleen or kidney; blurred hepatic vasculature; high attenuation of signals [21].

### Definitions for metabolic syndrome and cardiovascular risk factors

The diagnosis of metabolic syndrome was defined as having at least three criteria with the following cut points [22]: abdominal obesity (waist circumference ≥ 90th percentile of waist circumference of children of the same age and gender [23], elevated triglycerides (≥1.47 mmol/L [130 mg/dL]) [24], low HDL cholesterol (< 1.03 mmol/L [40 mg/dL]), elevated blood pressure [systolic blood pressure (SBP) ≥90th percentile of SBP or diastolic blood pressure (DBP) ≥90th percentile of DBP of children of the same age and height and gender], and impaired fasting glucose (≥5.6 mmol/L [100 mg/dL]). In addition to the factors of metabolic syndrome, insulin resistance was defined according to homeostasis model assessment of insulin resistance (HOMA-IR) which was calculated as fasting insulin (mU/mL) × [fasting glucose (mmol/L)/22.5] [25]. The insulin resistance threshold was defined as ≥3.16, as described in the literature [26]. Hyperuricemia was defined as uric acid value ≥357 umol /L [27].

### Statistical analysis

All statistical analysis was performed with SPSS version 25.0 software (SPSS Inc., Chicago, Illinois). Quantitative data with normal distribution were expressed as mean ± SD. One-way ANOVA was used to test differences among of the three groups, and multiple testing was analyzed with the least significant difference method. Quantitative data with non-normal distribution were expressed as median with interquartile range. The Kruskal-Wallis test was used to test differences among the three groups, and the Mann-Whitney U test was used for comparison between groups. Chi-square tests were performed for the differences in proportions, and odds ratio (OR) value and 95% confidence interval (CI) were calculated. Logistic dichotomous regression analysis was used to test NASH risk factors, and the adjusted OR and 95% CI were given. The significance level was defined at *p* < 0.05.

## Results

### Demographic and clinical characteristics of participants

A total of 428 children with obesity were recruited for the study, including 148 (34.6%) with SOB, 235 (54.9%) with SS and 45 (10.5%) with NASH. Anthropometric characteristics are shown in Table 1. There was no significant difference in age or Tanner stage classification among the three groups. Obesity indicators such as BMI, BMI-SDS, waist circumference, and Body fat in the NASH group were significantly higher than in the SS and SOB groups, but percentage of skeletal muscle in the NASH group was significantly lower than in the other groups (*p* < 0.001). Percentage of body fat and WHtR in the SS and NASH groups were significantly higher than in the SOB group, but there was no significant difference between the first two groups, indicating that these two anthropometric measurements could not well distinguish the spectrum of NAFLD.

**Table 1:** Comparison of anthropometric measurements

Groups	SOB	NAFLD		F/Z/χ^2^	*p-*value
		SS	NASH		
*N* (M/F)	148 (96/52)	235 (161/74)	45 (42/3)	13.73	0.001
Age (y)	10.19 ± 1.76	10.46 ± 1.71	10.65 ± 1.76	1.71	0.181
Tanner Stage, *n* (%)				4.38	0.357
1	86 (58.90%)	127 (55.70%)	23 (51.10%)		
2–3	53 (36.30%)	88 (38.60%)	22 (48.90%)		
4–5	7 (4.80%)	13 (5.70%)	0 (0.00%)		
BMI (kg/m^2^)	25.16 (2.79)^ab^	28.14 (3.93)^a^	29.45 (4.42)	39.75	0.001
BMI-SDS	2.72 (0.76)^ab^	3.20 (0.89)^a^	3.60 (0.98)	21.83	0.001
Body fat (kg)	20.50 (5.87)^ab^	26.67 (8.38)^a^	29.33 (10.29)	35.91	0.001
Percentage of body fat (%)	37.12 ± 5.38^ab^	40.40 ± 6.10	41.06 ± 6.27	16.15	0.001
Skeletal muscle (kg)	18.24 ± 4.53^ab^	20.58 ± 5.35	22.25 ± 6.99	13.03	0.001
Percentage of skeletal muscle (%)	33.01 ± 3.14^ab^	31.69 ± 3.43^a^	30.17 ± 4.78	11.64	0.001
Waist circumference (cm)	83.62 ± 8.48^ab^	92.50 ± 10.45^a^	96.14 ± 10.25	45.26	0.001
WHtR (%)	0.57 ± 0.05^ab^	0.61 ± 0.05	0.63 ± 0.04	35.42	0.001
SBP (mmHg)	111 ± 15^ab^	116 ± 16^a^	123 ± 16	11.86	0.001
DBP (mmHg)	65 ± 10^ab^	69 ± 10	71 ± 10	8.01	0.001

Normal distribution data are expressed as the mean ± SD. Non-normal distribution data are presented as median (interquartile). vs. NASH group, ^a^ *p* < 0.001; vs. SS group, ^b^ *p* < 0.001

*SOB* Simple Obese, *NAFLD* Nonalcoholic Fatty Liver Disease, *SS* Simple Steatosis, *NASH* Nonalcoholic Fatty Hepatitis, *M* Male, *F* Female, *BMI* Body Mass Index, *BMI-SDS* Body Mass Index Standard deviation score, *WHtR* Waist to height ratio, *SBP* Systolic blood pressure, *DBP* Diastolic blood pressure

### Comparison of biochemical parameters in children with obesity with or without NAFLD

The biochemical parameters are shown in Table 2. Children in the NASH group had higher hepatic enzymes including ALT and AST, indicating liver injury. The triglyceride levels were significantly higher in the NASH and SS groups compared with the SOB group. The uric acid concentration and HOMA-IR levels involved in the pathogenesis of NAFLD were ranked as follows: SOB < SS < NASH.

**Table 2:** Comparison of biochemical parameters between SOB and NAFLD

Groups	SOB	NAFLD		F/Z	*p-*value
		SS	NASH		
ALT (U/L)	17 (9)^ab^	26 (21)^a^	106 (41)	161.92	0.001
AST (U/L)	22 (8)^ab^	26 (14)^a^	62 (26)	112.41	0.001
Uric acid (μmol/L)	352 (96)^ab^	406 (124)^a^	455 (141)	49.96	0.001
Triglycerides (mmol/L)	1.05 (0.62)^ab^	1.37 (0.91)	1.31 (0.90)	16.83	0.001
Cholesterol (mmol/L)	4.25 ± 0.99^a^	4.33 ± 0.89	4.59 ± 0.88	2.29	0.103
HDL (mmol/L)	1.27 (0.37)^b^	1.17 (0.36)	1.18 (0.30)	18.23	0.001
Fasting glucose (mmol/L)	4.73 ± 0.44^b^	4.61 ± 0.48	4.61 ± 0.59	3.08	0.047
Fasting insulin (mU/L)	12.71 (12.08)^a^	15.01 (14.39)^a^	21.30 (7.60)	12.39	0.002
HOMA-IR	2.77 (2.53)^a^	3.06 (3.00)^a^	4.2 (4.61)	10.39	0.006

vs. NASH group, ^a^ *p* < 0.001; vs. SS group, ^b^ *p* < 0.001. Normal distribution data are expressed as the mean ± SD. Non-normal distribution data are presented as median (interquartile)

*SOB* Simple Obese, *NAFLD* Nonalcoholic Fatty Liver Disease, *SS* Simple Steatosis, *NASH* Nonalcoholic Fatty Hepatitis, *ALT* Alanine aminotransferase, *AST* Aspartate aminotransferase, *HDL* High-density lipoprotein, *HOMA-IR* Homeostasis model assessment of insulin resistance

### Prevalence of metabolic syndrome and cardiovascular risk factors in NAFLD compared with children without NAFLD

The occurrence rates of metabolic syndrome in the NASH and SS groups were significantly higher than in the SOB group (53.3, 49.8% vs 19.6%, *p* < 0.001). Children with NASH had significantly elevated blood pressure and triglycerides compared with children in the SOB group, but there was no difference in the frequency of abdominal obesity, low HDL and impaired fasting glucose when dichotomous cut points were used among groups (Table 3).

**Table 3:** Comparisons of metabolic risk factors between obese children with NAFLD and those without liver disorder

Factors	SOB (*N* = 148)	NAFLD		χ^2^	*p*-value
		SS (*N* = 235)	NASH (*N* = 45)		
**Abdominal obesity**					
Waist circumference (cm) ≥ p90, %	144 (97.3%)	234 (99.6%)	45 (100.0%)	4.67	0.097
**Elevated blood pressure**					
SBP or DBP ≥ p90, %	62 (41.9%)^a^	146 (62.1%)	30 (66.7%)	17.56	0.001
**Dyslipidemia**					
Triglycerides (mmol/L) ≥ 1.47 mmol/L, %	57 (38.5%)^ab^	133 (56.6%)	27 (60.%)	13.44	0.001
HDL (mmol/L) < 1.03 mmol/L, %	15 (10.1%)^b^	65 (27.7%)	8 (17.8%)	17.63	0.001
**Impaired fasting glucose**					
Fasting Glucose ≥5.6 mmol/L, %	2 (1.4%)	6 (2.6%)	2 (4.4%)	1.55	0.460
**Insulin resistance**					
HOMA-IR (≥3.16), %	57 (38.5%)^ab^	110 (46.8%)^a^	31 (68.9%)	12.87	0.002
**Hyperuricemia**					
Uric Acid (umol/L) ≥ 357umol/L, %	68 (45.9%)^ab^	161 (68.5%)^a^	39 (86.7%)	32.63	0.001
**Metabolic syndrome, %**	29 (19.6%)^ab^	117 (49.8%)	24 (53.3%)	38.47	0.001

vs. NASH group, ^a^ *p* < 0.001; vs. SS group, ^b^ *p* < 0.001

*SOB* Simple Obese, *NAFLD* Nonalcoholic Fatty Liver Disease, *SS* Simple Steatosis, *NASH* Nonalcoholic Fatty Hepatitis, *SBP* Systolic blood pressure, *DBP* Diastolic blood pressure, *HDL* High-density lipoprotein, *HOMA-IR* Homeostasis model assessment of insulin resistance

**Fig. 1:**
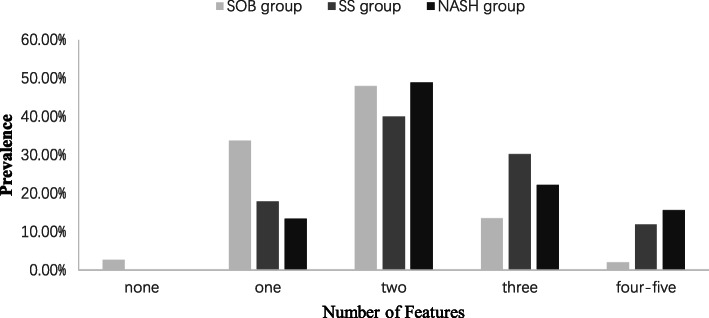
Features of metabolic syndrome in the three groups. Obese children with NASH or SS have significantly more features of metabolic syndrome compared to SOB (*p* < 0.001)

Further investigation indicated that the prevalence of insulin resistance (38.5% / 46.8% / 68.9%) and hyperuricemia (45.9% / 68.5% /86.7%) were steadily higher from the SOB group to the SS group to the NASH group, respectively (*p* < 0.001). The progression of NAFLD was positively associated with cardiovascular risk factors and metabolic syndrome in these children with obesity. As shown in Fig. 1, all children in the SS and NASH groups had at least one metabolic syndrome factor. Moreover, the distribution of metabolic syndrome features in children in the NASH and SS groups was significantly (*p* < 0.001) shifted to the right, with more features present than in children with obesity in the SOB group.

### Correlates of NASH with obesity or metabolic risks

After adjusting for all other variables among the children with obesity, the odds of having NASH were significantly higher in those with severe obesity (BMI-SDS ≥ 3) (*OR* 2.56; 95% *CI* 1.06–6.17) compared to those with mild obesity, males (*OR* 4.94; 95% *CI* 1.41–17.35) compared to females, those with hyperuricemia (*OR* 2.98; 95% *CI* 1.14–7.76) compared to those without hyperuricemia, and those with insulin resistance (*OR* 2.71; 95% *CI* 1.29–5.69) compared to those without insulin resistance (see Table 4).

**Table 4:** Logistic regression analysis of the correlates associated with NASH

	Adjusted OR (95% CI)	*p*-value
BMI-SDS category		
2 ≤ BMI-SDS < 3^r^	1	
BMI-SDS ≥ 3	2.56 (1.06–6.17)	0.037
Gender		
Females^r^	1	
Males	4.94 (1.41–17.35)	0.013
Hyperuricemia		
No^r^	1	
Yes	2.98 (1.14–7.76)	0.025
Insulin resistance		
No^r^	1	
Yes	2.71 (1.29–5.69)	0.009

Note: ^r^ Reference category. Variables included in the adjusted model were age, gender, Tanner stage, BMI-SDS category, abdominal obesity, elevated blood pressure, hyperuricemia, impaired fasting glucose, insulin resistance, low HDL and elevated triglycerides

*CI* Confidence interval, *OR* Odds ratio, *BMI-SDS* Standard deviation score for the body mass index

## Discussion

### Clinical characteristics of NAFLD in children with obesity

In the context of the obesity epidemic among children in China, the rate of NAFLD has become increasingly serious in the past 20 years. In this study of 428 children with obesity, 54.9% had NAFLD, and 10.5% NASH. Moreover, children with NAFLD had a higher risk of cardiovascular and metabolic syndrome than those without it. Severe obesity, gender, hyperuricemia and insulin resistance are risk factors for NASH in children with obesity. Clinical evidence indicates that children with NAFLD experience an increased incidence and mortality of cardiovascular disease in adulthood [9]. Early identification of the clinical characteristics is therefore particularly important for Chinese children with NAFLD and NASH in light of the current obesity epidemic.

Due to differences in diagnostic criteria, selected population and ethnicity, the reported prevalence of NAFLD varies greatly in the literature [3]. A meta-analysis of epidemiological studies in children showed that an average prevalence of NAFLD was 7.6% in general population studies and 34.2% in studies of children with obesity [3]. Due to the limitations of the invasive diagnostic methods for NASH, there is currently a lack of population-based studies on NASH. A recent community-based study reported that 193 subjects (55%) were diagnosed with NAFLD, and 105 subjects (30%) were diagnosed with NASH by biopsy [16]. In our study, the incidence of NAFLD in children with obesity was 65.4%, which was higher than other reports, and the incidence of NASH was 10.5%. The risk of NASH in severely obese (BMI-SDS ≥ 3) children was 2.56 times higher than in mildly obese (2 ≤ BMI-SDS < 3) children. These results highlight that obesity is still an important trigger of NAFLD, although NAFLD and NASH can be found in the normal BMI population.

At the same time, our results demonstrated that the incidence of NASH in males was significantly higher than in females (14% vs. 2.4%), and gender was still a risk factor for NASH after adjusting for age, obesity and other components of the metabolic syndrome (*OR* = 4.94, 95% *CI* (1.41–17.35)). The gender difference in the distribution of NASH is similar to the results in adults. However, the reasons for this are not clear so far. It is possible that the fat distribution is more likely to accumulate in the viscera in males, which is more likely to cause metabolic disorders compared with subcutaneous fat, and that estrogen can improve the insulin sensitivity of adipose tissue in females [28].

### NAFLD, cardiovascular risk and metabolic syndrome

In the present study, the NAFLD children showed higher cardiovascular risk and more metabolic syndromes than non-NAFLD children with obesity. This result is consistent to that reported in a previous case-control analysis [11]. The NAFLD children were more likely to develop abnormal glucose, insulin, triglycerides and HDL cholesterol than non-NAFLD children with matched age, sex and BMI [11]. A study covering 400 obese children also revealed that abnormal hepatic echo was associated with systolic and diastolic dysfunction [29]. Metabolic syndrome is a collection of cardiovascular risk factors that can predict diabetes and cardiovascular disease better than any single component. More and more evidence has shown that a large proportion of children with NAFLD meet the diagnostic standard of metabolic syndrome, suggesting that these children will develop diabetes and cardiovascular diseases in the future. This belief was supported by a 14-year-long Swedish cohort study of NAFLD patients diagnosed by biopsies. There was a 9% prevalence of diabetes at baseline, and most patients (78%) had developed impaired glucose tolerance or diabetes by the end of the 14-year period [18]. In the same study, the survival of NAFLD adults diagnosed by biopsy was lower than that of a matched control group, largely due to higher cardiovascular mortality [18]. Therefore, health care workers should pay more attention to the early identification of cardiovascular risk factors in the NAFLD population, which is helpful to facilitate targeted prevention and treatment strategies.

### NAFLD and insulin resistance

We found that the risk of insulin resistance in the NASH group was significantly higher than that in the SS and SOB groups, and insulin resistance was a risk factor for NASH after adjusting for confounding factors. Many clinical studies suggest that insulin resistance is always associated with NAFLD in adults and children [30–33]. A biopsy-proven NAFLD study showed that 95% of children had insulin resistance [34]. In an experimental study, Bugianesi et al. found that adipose tissue is an important site for the development of insulin resistance, which can contribute to NAFLD [35]. However, the association between insulin resistance and NASH remains unclear. The pathogenesis of NAFLD is hepatocyte steatosis and hepatocyte injury, in which insulin resistance plays an important role in the development of NAFLD. As peripheral insulin resistance arouses in NAFLD patients, serum-free fatty acids, synthesized through the lipolysis of visceral fatty tissue and dietary fat, serve as the origin of hepatic triglycerides. Insulin resistance acts in metabolic disorders induced by free fatty acids and mitochondria. Lipid overload can also repress oxidative performances. Meanwhile, with the production of reactive oxygen species, cytokines are induced, inflammatory cells chemoattracted, and hepatic stellate cells activated, all leading to damage to hepatocytes [36]. Therefore, insulin resistance is involved in the occurrence and development of NAFLD and NASH. On account of the central role of insulin resistance in glucose metabolism, youth diagnosed with fatty liver need further evaluation to exclude diabetes or impaired glucose tolerance due to insulin resistance.

### NAFLD and uric acid

A growing number of clinical studies have shown that hyperuricemia is related to NAFLD in both adults and children [37–39]. In a recent study of adolescents, hyperuricemia independently predicted [*OR* 2.5, 95% *CI* (1.87–2.83)] the presence of NASH after adjustment for potential confounders [40]. A biopsy-proven NAFLD study also found that the higher the serum uric acid, the greater the steatosis grade and lobular inflammation [41]. Similar to previous studies, we confirmed that obese patients with hyperuricemia had a higher risk of NASH occurrence [*OR* 2.98, 95% *CI* (1.14–7.76)]. Experimental studies observed that incubation of HepG2 cells with uric acid can increase intracellular triglycerides. The potential mechanisms may be that uric acid activates mitochondrial stress, subsequently activates endoplasmic reticulum stress and activates sterol regulatory element-binding protein 1C, while uric acid can stimulate the NOD-like receptor family pyrin domain containing 3 inflammasomes to regulate hepatic steatosis and inflammatory response in NAFLD patients [42, 43].

### Limitations of the present study

Firstly, we cannot confirm whether the presumed NASH patients do have abnormal hepatology, so we chose the combination of ultrasound and twice the upper limit of ALT to define NASH, in order to reduce the diagnostic error. Secondly, a total of 428 obese children were included in this study, nearly 70% being males. Thirdly, because of its cross-sectional nature, the causal relationship between NAFLD and cardiovascular risk factors was not fully explored. In addition, other potential factors such as dietary, lifestyle, and family genetic factors will be taken into account in future studies.

## Conclusion

In this study, we investigated the clinical characteristics of NAFLD and NASH in children with obesity, as well as the associations between NAFLD and metabolic risk factors at a single center. Our findings indicated that the prevalence of NASH in children with obesity is associated with high BMI-SDS, gender, insulin resistance, and hyperuricemia. These findings highlight the need for monitoring and prevention strategies at an early stage for children with obesity. Further multi-center and prospective epidemiological studies are needed to determine the prevalence and development of NAFLD in children with obesity in China.

## Data Availability

The data used to support the findings of this study are available from the corresponding author upon request.

## Abbreviations

ALT: Alanine transaminase
AST: Aspartate aminotransferase
BMI: Body mass index
BMI-SDS: Body mass index standard deviation score
CI: Confidence interval
DBP: Diastolic blood pressure
F: Female
HDL: High-density lipoprotein
HOMA-IR: Homeostasis model assessment of insulin resistance
M: Male
NAFL: Nonalcoholic fatty liver
NAFLD: Nonalcoholic fatty liver disease
NASH: Nonalcoholic fatty hepatitis
OR: Odds ratio
SBP: Systolic blood pressure
SOB: Simple obesity
SS: Simple steatosis
WHtR: Waist to height ratio
